# Observations on the Structure and Use of the Thyroid Glands in the Horse, and on Some Diseases of That Animal

**Published:** 1821-04

**Authors:** James White

**Affiliations:** Late Veterinary Surgeon of the 1st or Royal Dragoons; and Veterinary Surgeon to the Bath and West of England Agricultural Society.


### *Observations on the Structure and Use of the Thyroid Glands in the Horse, and on some Diseases of that Animal.* By James White, late Veterinary Surgeon of the 1st or Royal Dragoons; and Veterinary Surgeon to the Bath and West of England Agricultural Society.

IT had already been supposed, by several anatomists*—from analogy founded on its structure, situation, and the nature of the fluid found in its cells,—that the thyroid body is a mucous gland, and that its excretory ducts open into the larynx or trachea; but the truth of this inference had not hitherto been demonstrated by any positive evidence.† I have, how-

* See Galen, *De usu Partium;* and Morgagni, *Advers. Anat.* t. iii. for a full account of the opinions of anatomists on this subject up to the early part of the last century.

† Cuvier acknowledges (in his Lectures on Comparative Anatomy) that we are ignorant of its functions; and the only remarks he makes at all relative to this point tend to show that it exists exclusively in the *mammalia*, and is present in all the animals of this class excepting the *cetaceæ*. Its absence in animals of this family, which appear to be devoid of voice, has led to an inference that it is connected with this function; but this inference is somewhat opposed by the fact that it is present in the porpus, which is not known to possess the power of voice.

ever, satisfactorily ascertained, by means of injections with ink, that it is a mucous gland, and that its excretory ducts open into the larynx: the termination of them may be readily seen as minute papillary eminences, especially on the epiglottis and superior parts of the larynx. The excretory ducts are continued down over the rima glottidis into the trachea, especially at its posterior part, where the bronchial membrane is thrown off from the cartilages, and the intervening space is filled up with cellular membrane.

These excretory ducts communicate with the cells in the substance of the thyroid gland, (or, more properly speaking, *glands;* for it is obviously constituted of two glands united together by a band of dense cellular texture. The division is very evident in several of the ape tribe, and in bats the two are completely separate;) and hence it is, as I shall presently show, that diseases of this gland often lead to troublesome disorders of the respiratory organs.* The secretion of those glands sometimes becomes too abundant, and is also morbid in its qualities, which, being poured into the larynx, produces irritation there, and not unfrequently ulceration, especially in the edges of the rima of the glottis; and hence the worst kinds of *roaring* arise.

---

* There are several cases on record that seem to show that this is equally the case in regard to the human body. Before I notice these, I should remark that it is an extraordinary circumstance that the observations of Bordeu on this subject have been so little attended to by anatomists. Bordeu says (in his *Recherches Anatomiques sur les Glandes*, § xlii.) that the first ring of the trachea is always perforated by one, two, or three foramina, about the middle of its anterior surface. This was first observed by him on dissecting a larynx before a fire: on the thyroid gland, which was extremely large, being removed, he found the ring above mentioned almost osseous, but sufficiently diaphonous to permit him to perceive, by means of the fire, two foramina, which were covered by fine membranes, easily separable. These foramina, he felt satisfied, existed naturally in the cartilage: they were situate one on the side of the other, were of an oval shape, and of about a line in extent in their shortest diameter. After this, he always discovered openings of this kind, most ordinarily two, though sometimes one and sometimes three were found to be present. They are, he remarks, very different from the small irregular holes dispersed here and there in the trachea and thyroid cartilage; and he inferred, from their situation, that they were connected with the thyroid glands. That such was the case, he became more confident some time afterwards, on dissecting the body of a person who had suffered a violent death. On examining the internal surface of the trachea, in the situation above described, without having touched the thyroid gland, he found the membrane lining it full of small holes; and on passing bristles, lightly, through five of those openings, they assembled together, three on one side and two on the other, in the openings in the ring of the trachea above mentioned; and, continuing to urge them forwards, in a gentle manner, they passed into the substance of the thyroid gland. Lamure, of Montpellier, was present on this occasion. The same results were witnessed in several experiments on other subjects. He also says, that the thyroid gland was inflated by blowing through those openings. I have been informed that Mr. Copland Hutchison (in a paper lately read to the Medical and Chirurgical Society, on the Cure of Bronchocele by Seton,) has stated that Mr. Fodere has mentioned a communication between the larynx and this gland, which was inflated by blowing air into the larynx properly secured by ligatures; but, whether the inflation was produced through some communicating

The disordered secretion of the thyroid glands has appeared to originate from improper feeding, especially with bad hay, or such as is made too late,—that is, after the seed has formed. The tender shoots with which grass abounds at the proper period for mowing, and which are richly supplied with saccharine juices, become then tough and fibrous, and the saccharine juice that is not directed to the seed-stalks is converted into fecula and neutral salts. Such hay, then, abounds with materials for forming acrimonious secretions, and is greatly deficient in saccharine or nutritious matter. Besides this, such hay is apt to produce a depraved and inordinate appetite, by which the horse is led to eat to excess; and hence additional means are established for the production of disorders of the respiratory organs: for the secretion of the whole of the mucous membrane of the bronchial tubes and *air-cells*,—as they are improperly named, for they are really tubes, though capable of being dilated in certain parts by injections or quicksilver, and of being made to assume an appearance which has led to this erroneous term for them,—as well as of the thyroid gland, becomes too abundant, and probably also morbid in quality; and thus some are plugged up and obliterated, while others are distended and ruptured, and the air is effused into the cavity of the thorax. These consequences, with a debilitated state of the muscles of respiration, are the causes of those symptoms called *broken-wind*, which may be produced solely by the improper use of hay, and especially of bad hay. It is commonly immediately excited by the immoderate exertions to which the animal is impelled at a time when the stomach and bowels are loaded with food and excrements and the air-vessels replete with mucus.

Having noticed the ill qualities of bad hay, that is, hay made after the seed is formed, it may be expected that I shall say something of good hay, that is, hay which is mown when the grass is breaking out into flowers: (which is generally in the beginning of June:) such grass abounds with young and tender shoots, full of saccharine juices, and, when dried and properly fermented in the mow, it is of a green colour, and contains various grasses, commonly called herbage. This is the hay, and this only, which should be given to horses and cattle.

19, *Vineyards*, *Bath*; *Feb*. 7, 1821.

---

ducts, or that the air insinuated itself into the cellular substance of the part, does not appear. He recommends that examinations of the parts in cases of monstrosities and disease, should be made, as the structure of parts has sometimes been discovered by this means, when, from their minuteness, they, in the natural state, elude our researches. Borden, in regard to this view of the subject, mentions a case in which an abscess of this gland was suddenly evacuated through the trachea, without any previous disorder in the respiration of the patient; another, where mucous matter, in great abundance, was expectorated, and which appeared to come from the same source; and several others, tending to establish, more or less generally, the same inferences.

